# Comprehensive Multi-Analytical Investigations on the Vietnamese lacquered Wall-Panel “The Return of the Hunters” by Jean Dunand

**DOI:** 10.1038/s41598-019-55471-6

**Published:** 2019-12-11

**Authors:** Valentina Pintus, Anthony J. Baragona, Karin Wieland, Michael Schilling, Silvia Miklin-Kniefacz, Christoph Haisch, Manfred Schreiner

**Affiliations:** 1 Institute of Science and Technology in Art, Academy of Fine Arts Vienna, Schillerplatz 3, Vienna, A-1010 Austria; 2 Institute of Art and Technology, Department of Conservation Science, University of Applied Arts, Salzgries 14, A-1010 Vienna, Austria; 3 Chair of Analytical Chemistry, Institute of Hydrochemistry, Technical University of Munich (TUM), Marchioninistraße 17, D-81377 Munich, Germany; 4 Getty Conservation Institute (GCI), Los Angeles, CA USA; 5 Bernardgasse 4/1, Vienna, A-1070 Austria

**Keywords:** Infrared spectroscopy, Characterization and analytical techniques

## Abstract

This work presents a comprehensive, multi-analytical scientific approach for determining the type of lacquer and artistic materials used by Jean Dunand on his work “The Return of the Hunters” (1935). For this purpose, thermally assisted hydrolysis and methylation – gas chromatography/mass spectrometry (THM-GC/MS), optical microscopy (OM) in visible (Vis) and ultraviolet light (UV), and scanning electron microscopy - energy-dispersive X-ray spectroscopy (SEM-EDX) were selected. Furthermore, a novel application of micro attenuated total reflection Fourier transform infrared (µATR-FTIR) spectroscopic mapping by univariate and multivariate analysis was applied for studying the complex lacquer paint stratigraphy. The results show that Vietnamese lacquer was used as a binder, mixed together with linseed oil and pine resins as additives in combination with inorganic pigments, and that shellac was included on the top of the paint; they document an important step in the story of the transfer of Vietnamese lacquer painting techniques to Europe.

## Introduction

### “The Return of the Hunters” by Jean Dunand as an example of Vietnamese Lacquer in Europe

**Figure 1:**
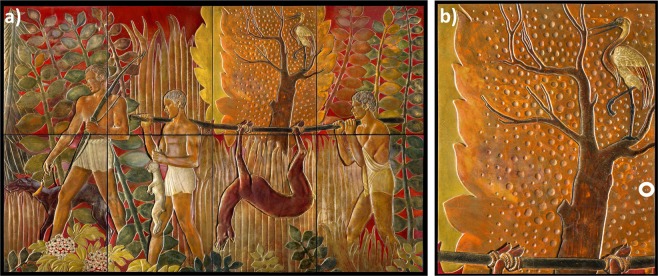
(**a**) “The Return of the Hunters” (1935) by Jean Dunand based on eight panels and (**b**) the third panel at the top from the left side subject of the investigations, showing the sampling area as a circled area on the lower right side. Photos by Renate Neder.

During the 1920s and 1930s extensive contact between European and Asian Cultures encouraged the exchange of their respective artistic knowledge. The founding of the Ecole des Beaux Art in Hanoi in 1925 by the artists Victor Tardieu and Nam Son facilitated this exchange, with the use of traditional Vietnamese lacquer as a painting medium applied to a flat-bidimensional surface^1^ and subsequent exportation of this lacquer to Europe from the French colonies being one of the results of this fruitful collaboration. One of the artists fascinated by Asian lacquer techniques was Jean Dunand. He was born on the 20th of May, 1877 in Lancy, Switzerland and became one of the most well renowned French Art Deco artists of his time. He mastered Asian lacquer techniques and combined them with modern Art Deco designs to decorate furniture, wall paneling, and metal vases^2^. He studied sculpture at the Geneva School of Industrial Arts until 1897 when he moved to France, continuing his studies at the Ecole des Beaux Art. Dunand became a master of furniture and decorative object design, and subsequently became interested in Asian lacquer techniques, which he started studying at the beginning of the 20th century. In 1912 he undertook an intensive two-month training session in the usage of Asian lacquer techniques under the tutelage of the famous Japanese lacquer teacher Seizo Sugawara. After World War I, Jean Dunand decided to open a lacquer studio in his workshop, where the lacquer used for his artworks was mainly obtained from the French colonies in Indochina, and most of his craftsmen were also Indochinese^2^. The 1920s and 1930s were Dunand’s most successful and creative years characterized by lacquer as integral element of his artistic oeuvre. The 8-piece wall panel “The Return of the Hunters” (1935 – total ≈6 × 5.80 m, Fig. 1) from the original 24-piece wall panel “The Hunt” was a creation from that period. All 24 panels were originally used for furnishing the interior of the smoking room of the luxurious transatlantic cruise ship *Normandie* until 1941. Successively the panels were moved to different locations, including a storehouse of the Compagnie Générale Transatlantique (CGT) in New York, where they were kept until 1950. Later they were reduced in size and modified and then used in another French cruise ship named *Liberté* from 1950 to 1962. Finally they were sold in several lots in Le Havre^3^. One lot was the 8 panels of “The Return of the Hunters”, which were again reworked and subjected to reduction in size at some time before 1992. In this year the eight panels were auctioned at Sotheby’s in New York and subsequently transported to Vienna, where they were installed on the wall of a private residence^3^. The other panels of “The Hunt” ended up in different places, such as Carnegie Museum of Arts (Pittburgh, USA), Musée des Arts Décoratifs (Paris, France), Ecomusée (Saint Nazaire, France), and in the Musée d’Art Moderne de la Ville de Paris (Paris, France)^3^.

To the knowledge of the authors the wall-panel “The Return of the Hunters” by Jean Dunand represents one of the first examples of the use of Vietnamese lacquer as a painting medium in Europe, and is therefore of great historical and artistic importance.

### The scientific investigation of Asian lacquers

Asian lacquers have been increasingly investigated scientifically over the last decade^4–14^, not only when used as decorative coatings for wood, ceramics, and metal objects but also as a painting medium. These investigations have mostly focused on determining their botanical origin, because the identification and characterization of their precise chemical composition is essential for developing appropriate and meaningful conservation-restoration strategies. While all Asian lacquers come from trees of the *Anacardiaceae* family, the chemical composition of the lacquer produced is slightly different from species to species. The lacquer used in China, Japan and Korea, known as urushi, is taken from the *Rhus vernicifera* species; that used in Thailand, Burma, and Cambodia, named thitsi, is extracted from the *Melanorrhea usitate*, while the lacquer used in North Vietnam and Taiwan, called laccol, is obtained from the *Rhus succedanea*. These three species produce a sap composed in the greatest proportion of phenol derivatives better known as catechols (60–65%), followed by water (30%), plant gum (7%), water-insoluble glycoproteins (2%), and the laccase enzyme (1%), which is responsible for activating the polymerization reaction during the drying process. The catechols of laccol are mainly formed by a side aliphatic chain composed of a maximum of 17-carbon atoms (3-heptadecylcatechol), while those of urushi are characterized by 15-carbon atom compounds (3-pentadecylcathecol). Thitsi contains alkyl benzenes (alkylphenylphenols and alkylphenyl-1,2-dihydroxybenzenes) in addition to the typical compounds of laccol and urushi. Vietnamese lacquer as a painting medium is also commonly mixed with drying oils (linseed, tung, perilla, sesame and rapeseed^15^) for increasing the gloss of the dry film^1^. This can occur during their manufacture, but is also done by the artists, who additionally often mix in pine resin to modify the workability of the lacquer. Furthermore, colored lacquers were and continue to be made by the addition of pigments such as vermilion and iron oxide for red, cadmium and chrome yellow for yellow, Prussian blue for blue, barium sulfate, zinc and lead oxides, crushed eggshell, and bone white for whites. Green-colored lacquer was created either by the use of chromium oxide pigment or by mixing yellow and blue pigments^2^. Black and brown lacquers are obtained by processing the lacquer at different temperatures, rather than the addition of mineral ingredients^1^.

Analytical investigations are crucial in order to characterize and identify Vietnamese lacquer chemically, and thus to distinguish it from urushi and thitsi. While many scientific studies have focused predominantly on the best known and most widely employed urushi lacquer, only one publication specifically dealing with Vietnamese lacquer has been reported to date^1^. Thermally assisted hydrolysis and methylation of pyrolysis-gas chromatography/mass spectrometry (THM-GC/MS) is currently the most suitable method for characterizing the widest range of organic materials included in the Asian lacquer as well as of its additives^4^, such as drying oils and natural resins. In comparison to the conventional pyrolysis-gas chromatography/mass spectrometry (Py-GC/MS), THM-GC/MS allows the identification of aged and oxidized compounds such as acid catechols and the detection of carbohydrate markers that originate from the gum and glycoprotein content^4^ in addition to the main pyrolysis compounds of the *Anacardiaceae* lacquers.

### Aim of the research

In this work, scientific investigations were performed on samples acquired from the wall-panel “The Return of the Hunters” by Dunand with the aim of informing conservation-restoration strategies. Since it was known that the panels were subjected to several previous modifications^3^, the main goal of the restoration campaign was to conserve *all original layers* and thus the understanding of their main chemical composition was fundamental. Old retouching work and overpainting would be left as found, reduced where possible and/or adjusted in terms of coloration. For this purpose, it was important to sample an area for scientific investigations that had no overpainting to establish the original stratigraphy. The 8 panels were examined with a hand-held UV light, which quickly showed which areas had been overpainted due to the distinctive fluorescence of the overpainting material. The third panel on the top from the left side (Fig. 1) showed no overpainting and was therefore selected as the area that would be sampled for further analysis, the results of which make up the body of this work. The main goal of the analyses was to reveal the paint stratigraphy, which type of lacquer Dunand used, and additionally to detect the possible presence of organic additives. Unfortunately, sampling was limited to this area only due to financial restrictions.

For this study thermally assisted hydrolysis and methylation-gas chromatography/mass spectrometry (THM-GC/MS) was employed for determining the lacquer type. Additionally, optical microscopy (OM) in visible (Vis) and ultraviolet (UV) light, scanning electron microscopy-energy-dispersive X-ray spectroscopy (SEM-EDX) were used for investigating the lacquer paint stratigraphy and inorganic components included, such as pigments. Furthermore, a novel application of micro-attenuated total reflection of Fourier transform infrared (µ-ATR-FTIR) spectroscopic mapping by univariate and multivariate analysis was employed to investigate the spatial distribution of the lacquer in the painting’s cross-section as well as its additives detected with THM–GC/MS, thus assigning each component to a specific layer.

## Results and Discussion

### Examination of the lacquer technique by optical microscopy (OM) and scanning electron microscopy-energy dispersive X-ray spectroscopy (SEM-EDX)

**Figure 2:**
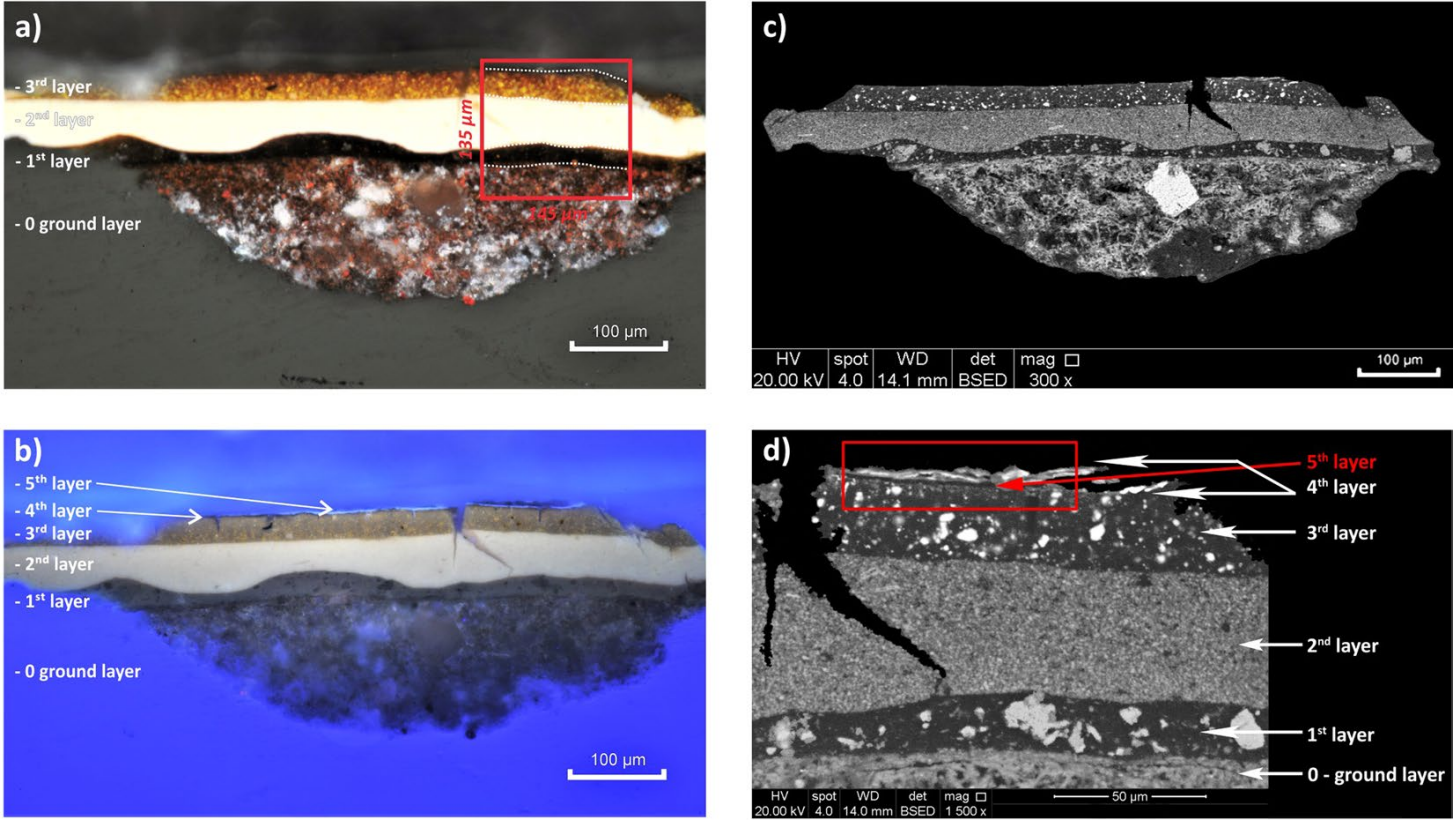
(**a**) Optical microscopy image of the cross-sectioned sample observed by reflected visible light showing the main layers as well as the µATR-FTIR mapped area (red box - Section 2.3) and (**b**) by ultraviolet light. (**c)** SEM micrograph of the whole cross-sectioned sample and **(d)** detail of the area investigated by point and bulk EDX analysis showing the main lacquer paint layers.

To allow cross-section investigation, the sample P3_L was embedded in polyester and epoxy resin before the dry surface was polished with abrasive grinding paper (for details see Supplementary Information). Examination of the cross-sectioned, stratigraphic sample by OM (Fig. 2a,b) and SEM-EDX (Fig. 2c,d and Table 1) revealed the favored “*Laque arrachee*” technique of the Dunand workshop, which consisted of lifting the fresh lacquer with a wooden spatula to create an uneven surface^2^. This was similarly used for one of the most impressive interiors realized by Dunand in 1928 for the penthouse of Templeton Crocker in San Francisco as well as for a series of pictorial wall panels entitled “Les peoples d’Asie et d’Afrigue” made for the 1931 Exposition Coloniale in Paris^2^. In this case the “*Laque arrachee*” consists of a black, compact and homogeneous (≈20 µm thick) layer (Layer 1) with a wavy surface applied on a ground layer (Layer 0) which only contains a few small, dispersed greyish grains of silica and feldspar as the inorganic components (Fig. 2). The reddish-greyish ground Layer 0, with an average thickness of ≈135 µm, is mostly characterized by gypsum plaster (CaSO_4_ • 2H_2_O - Ca and S in a ratio ≈1:1). This is visible in SEM as an open matrix of hydrated gypsum, as well as by OM and SEM as dark-brown or greyish un-hydrated particles. Layer 0 additionally contains unevenly distributed red grains, visible by OM and identified by EDX point analysis as iron (Fe). This suggests that Dunand colored the ground layer red in order to conceal the nature of the white plaster ingredients, a practice reported elsewhere^16^. The black Layer 1 was followed by the application of a homogeneous layer (Layer 2) with a pale yellow color, ≈40 µm thick, containing both of titanium oxide (TiO_2_) and bone white (Ca_3_(PO_4_)_2_) grains.

**Table 1:** Elements detected by bulk EDX analysis per layer on the cross-sectioned sample.

Layer	Elements
4	Au* (+++), Cu** (+++), S (++), Ti (+), Ca (+)
3	**S (+++), As (++), Hg (trace)**, Na (++), Ti (++), Si (+), Al (+), Cu (+)
2	**Ti (+++), Ca (++), P (+)**, S (++), Si (++), As (+), Al (+), Fe (+), Na (+), K (trace)
1	**Si (+++), Al (++)**, Ca (+), Ti (+), Fe (+), Mg (+), S (+)
0	**S (+++), Ca (+++), Fe (+)**, Si (+), Al (+)

Main elements are bold written. (+++ high, ++ moderate, + low concentration). *left side and **right side of the main crack in Fig. 2.

Above this, Layer 3 is observable by visible light as a dark orange layer (≈25 µm). It is formed by the arsenic-based pigment orpiment (As_2_S_3_). Analysis of numerous small orange grains by EDX point analysis, as well as the layer bulk EDX analysis of the entire layer (shown in Table 1) confirms an As to S ratio of 2:3, corresponding with orpiment. This layer furthermore contains a small quantity of the red pigment vermillion (HgS) detectable by point EDX analysis. Ultraviolet light optical microscopy showed two additional very thin uppermost layers (Fig. 2b), one of which appears black due to the absence of fluorescence (Layer 4) and another, which exhibits strong bluish fluorescence (Layer 5). This latter one is likely ascribable to a coating/protective layer, which was further investigated by µATR-FTIR (Section 2.3). The layer that appears black under UV irradiation is composed of gold on the left side of the sample and copper on the right side, leading to the interpretation that Dunand used both of these two metals for imparting different finishing color effects to the paint surface. In Fig. 2d, this metallic layer was detached during sample preparation and appears both above and below Layer 5 and therefore labelled as “folded up Layer 4”. This sample was taken from an area of the panel visibly characterized by a bi-metallic appearance, very likely due to the application of strewn or sprayed gilding décor, which is reported elsewhere as a technique that Dunand used, along with the application of gold leaf or a bronze finish^3^. Sugawara also taught the incorporation of metal leafs and powder as a gilding technique to Dunand during the 1912 lacquer training session^2^.

### Insight into the complex material composition: THM-GC/MS results

**Figure 3:**
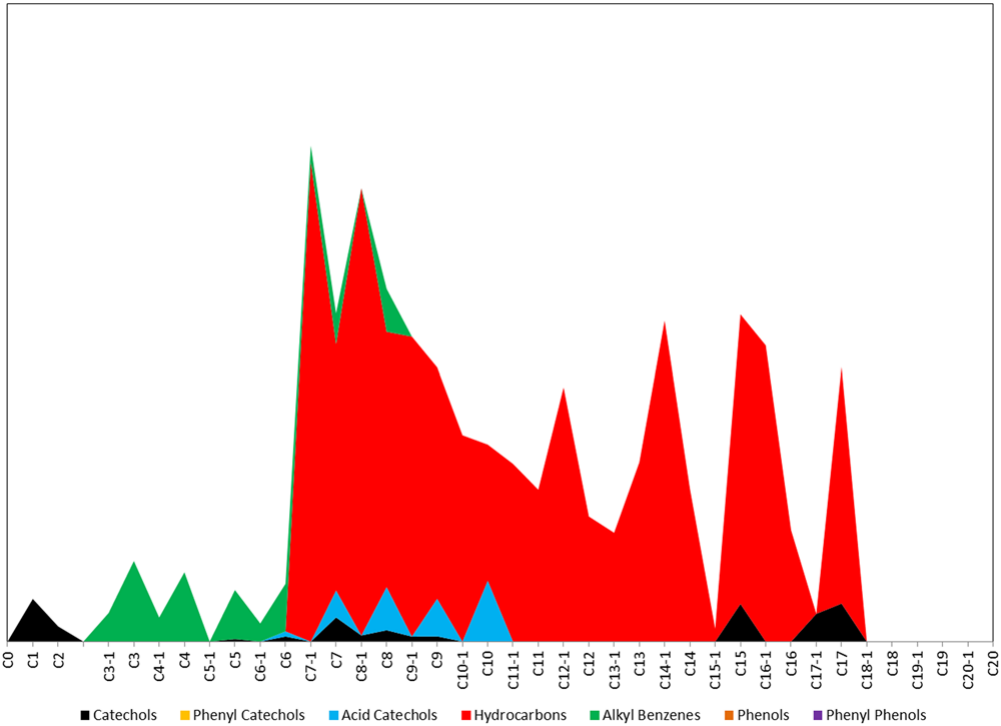
Gestalt graph of Anacard markers detected in the investigated paint sample.

The THM-GC/MS results highlight the complex composition of the different types of material that Dunand used for his lacquer panel painting. Firstly and mostly importantly, the presence of the Vietnamese lacquer laccol was evidenced by the identification and characterization of several characteristic *Anacardiaceae* tree sap pyrolysis compounds. The stacked area “gestalt” graph shown in Fig. 3 shows each specific *Anacardiaceae* tree sap pyrolysis compound (e.g.cathecol, acid cathecol, alkyl benzene etc.) in one color by plotting the peak area in the y-axis of the number of carbon atoms and double bonds in the x-axis^4^. Those detected are represented by acid catechols, with C_10_ arlenic acid as the most abundant, catechols with a maximum side chain length of C_17_ and aliphatic hydrocarbons with C_7_ as the most intense ones, and a series of alkyl benzenes from C_3_ to C_10_.

**Figure 4:**
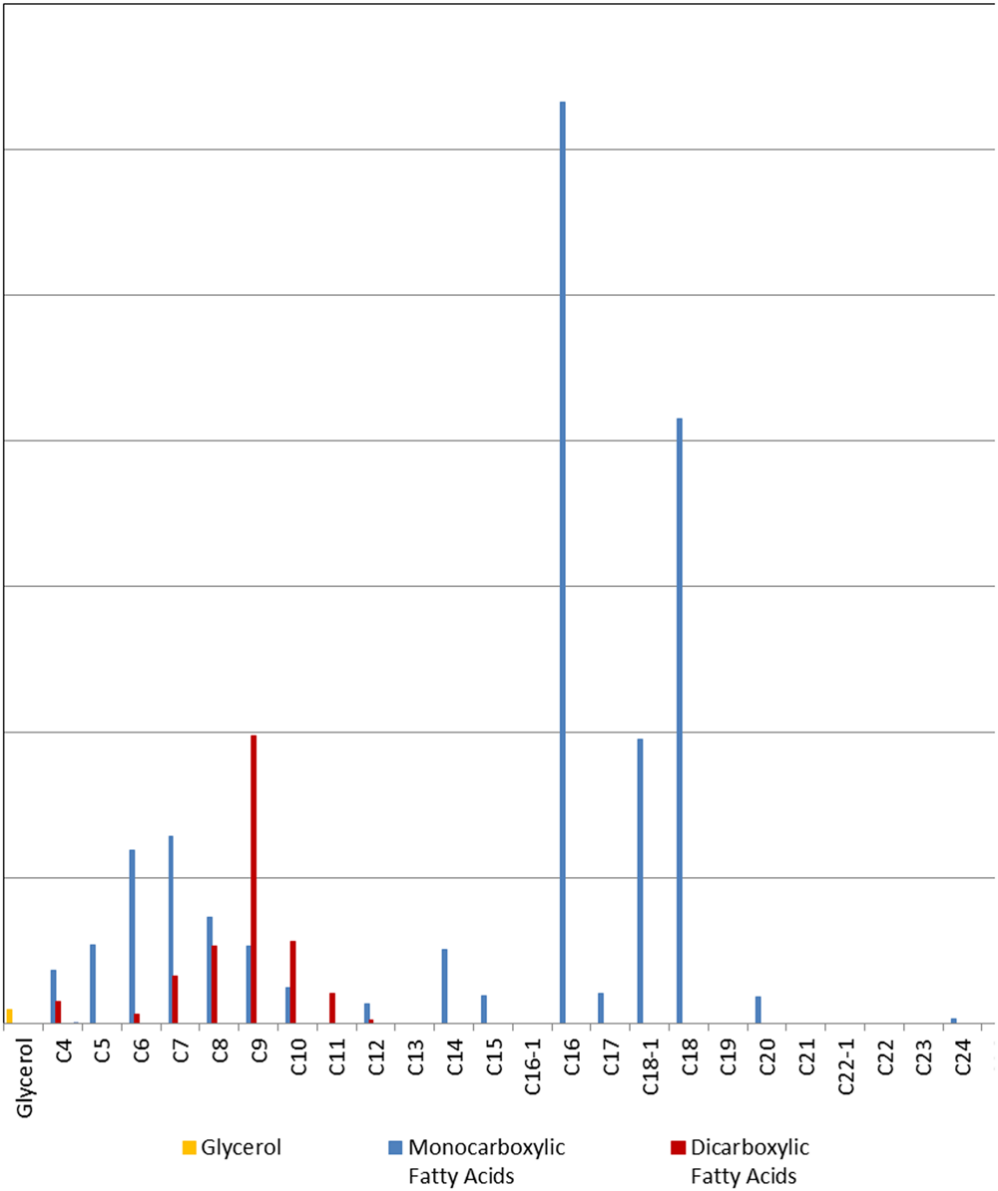
Fatty acid bar graph for the investigated paint sample.

Another indication of the presence of laccol was the detection of some carbohydrate compounds (Table 2) originating from gums and glycoproteins. These occur naturally in Anacard saps, and are almost three times higher in laccol than urushi sap^17^. They are represented by a group of three compounds eluted at about 1500 RI units with base peak of *m/z* 129 and another one found at 2467 RI units with *m/z* 88. The first set was probably formed during pyrolysis from monosaccharides and uronic acids, while the second one was mainly generated from the pyrolysis of disaccharides^4^. In addition to laccol, the use of a drying oil was suggested by the detection of both monocarboxylic (e.g. C_16:0_ palmitic, C_18:1_ oleic, C_18:0_ stearic acids) and dicarboxylic acids (e.g. C_8:0_ suberic, C_9:0_ azelaic, C_10:0_ sebacic acids) as well as by glycerol, which is shown by the bar graph in Fig. 4. Based on the palmitic to stearic acid ratio P/S of 1.5, linseed oil was identified as the type of drying oil found in the paint sample.

**Table 2:** List of the target compounds for specific groups detected by THM-GC/MS, with their corresponding compound name, Retention Index (RI), and mass to charge ratio (m/z).

Group type	Target compound name	RI	m/z
Carbohydrate - Anacards	Laccol carbohydrate - unverified 3	1501	75, 101, **129**, 161, 191
	Laccol carbohydrate - unverified 4	1526	75, 101, **129**, 161, 191
	Laccol carbohydrate - unverified 6	1554	75, 101, **129**, 145, 161
	Laccol carbohydrate - unverified 10	2467	75, 88, 101, **129**, 219
Resin - *Pinaceae*	6-dehydroabietic acid, methyl ester	2377	141, 165, 197, **237**, 312
	Dehydroabietic acid, methyl ester	2383	173, 197, 239, 299, **314**
	Mercusic acid, dimethyl ester	2456	**121**, 181, 304, 305, 364
	7-methoxy-tetra-dehydroabietic acid, methyl ester	2481	227, 267, 282, 327, **342**
	7,15-dimethoxy-tetra-dehydroabietic acid, methyl ester	2611	297, 313, 340, 357, **372**
	7-oxo-dehydroabietic acid, methyl ester	2624	187, **253**, 269, 313, 328
Resin - Shellac	Laccishelloic acid: dimethyl ester, methyl ether	2119	167, **262**, 275, 291,322
	Jalaric acid, tetramethyl (Ken)	2166	45, **75**, 291, 304, 336
	Jalaric acid, tetramethyl (Henk)	2206	45, **75**, 291, 304, 336
	Shelloic acid: dimethyl ester, dimethyl ether	2246	201, 228, 261, 289, **320**
	Aleuritic acid methyl ester, trimethyl ether	2375	71, **95**, 137, 159, 201
Protein - Animal glue	1H-Pyrrole, 1-methyl	736	39, 43, 53, 80, **81**
	Pyrrole	754	39, 41, **67**
	2-aminopyridine	1017	39, 42, 51, 67, **94**
	Protein 3 - blood & glue	1702	42, 70, **83**, 112, 168
	Glue marker - Mazzeo	1740	65, 93, 130, **186**
Pigment - Arsenic based	Dimethyl-methylthio-arsine	842	89, 109, 121, **137**, 152
	Arsenic -As_4_	1379	75, 150, 225, **300**
Various	Dimethyl disulfide	749	45, 46, 61, 79, **94**
	Dimethyl sulfate	868	45, 66, **95**, 96, 125
	Trimethyl phosphate	944	79, 95, 109, **110**, 140

Besides laccol and linseed oil, characteristic marker compounds of a diterpenic resin such as that from *Pinaceae* (pine) tree were also detected (Table 2). Those compounds are mainly characterized by dehydroabietic acid (DHA)-methyl ester, 6-DHA-methyl ester, 7-methoxy-tetra-DHA-methyl ester, 7-oxo-DHA-methyl ester, and 7,15-dimethoxy-tetra-DHA-methyl ester, which are oxidation products of the main diterpenic resin component abietic acid. The additional detection of the mercusic acid-dimethyl ester and the absence of palustric acid may indicate a pine resin obtained from the *Pinus latteri* species, which is native to Southeast Asia including Vietnam as well as southeast Burma, north Thailand, Laos and Cambodia^1,18^.

Other information about the complex sample composition was highlighted by the presence of some protein diagnostic markers (1H-pyrrole,1-methyl, pyrrole, 2-aminopyridine, protein 3-blood & glue, and glue marker-Mazzeo) denoting the presence of animal glue (Table 2), also complemented by the detection of dimethyl disulfide and dimethyl sulfate (Table 2), which are normally produced by sulfur containing amino acids under the analytical conditions employed in this study. In addition to Vietnamese lacquer and the organic additives linseed oil and pine resin, the THM-GC/MS investigations on the uppermost layers of the panel painting revealed the presence of shellac, also known to have been widely used by Dunand^2^. The typical hydroxyl aliphatic and terpene acids derivatives of shellac such as aleuritic acid derivatives, shelloic and laccishelloic acids, as well as jalaric acids^19^ were identified (Table 2).

Beyond the identification of organic compounds, hints of the presence of the two inorganic pigments orpiment (As_2_S_3_) and bone white (Ca_3_(PO_4_)_2_) can be derived from the THM-GC/MS results (Table 2). Arsenic based pigment was shown by the detection of dimethyl-methylthio-arsine (C_3_H_9_AsS) pyrolysis product and of its pyrolysis product As_4_, while bone white was detected by the presence of trimethyl phosphate, which is normally formed when bone black and the phospholipids in blood and egg are pyrolyzed under the conditions used in this study^2^. These results regarding the inorganic pigments used in this work correspond with the SEM-EDX analysis discussed in Section 2.1.

### Towards the assignment of each investigated organic compound to the paint stratigraphy

The final step of the analysis had the goal of assigning each individual organic compound detected in the paint sample by THM-GC/MS, such as the Vietnamese lacquer and its additives, linseed oil, pine resin, and shellac, to a specific stratigraphic layer of the polished cross-sectioned sample. This task is particularly important for a better understanding of the painting’s stratigraphy, and therefore the artistic technique used by Dunand. For this purpose, µATR-FTIR measurements were performed on a representative sample area that included all fundamental paint layers (red box in Fig. 2a). The overall area of analysis corresponded to 145 × 135 μm^2^, with 10 × 10 points of measurements, resulting in a total of 100 spectra. Considering a numerical aperture of 0.6, an effective sampling area of ≈20 × 20 μm^2^ can be estimated^20^. With a step size of 16 μm in the x direction and 15 μm in the y direction, a reasonable oversampling during the mapping was achieved to increase image contrast.

**Figure 5:**
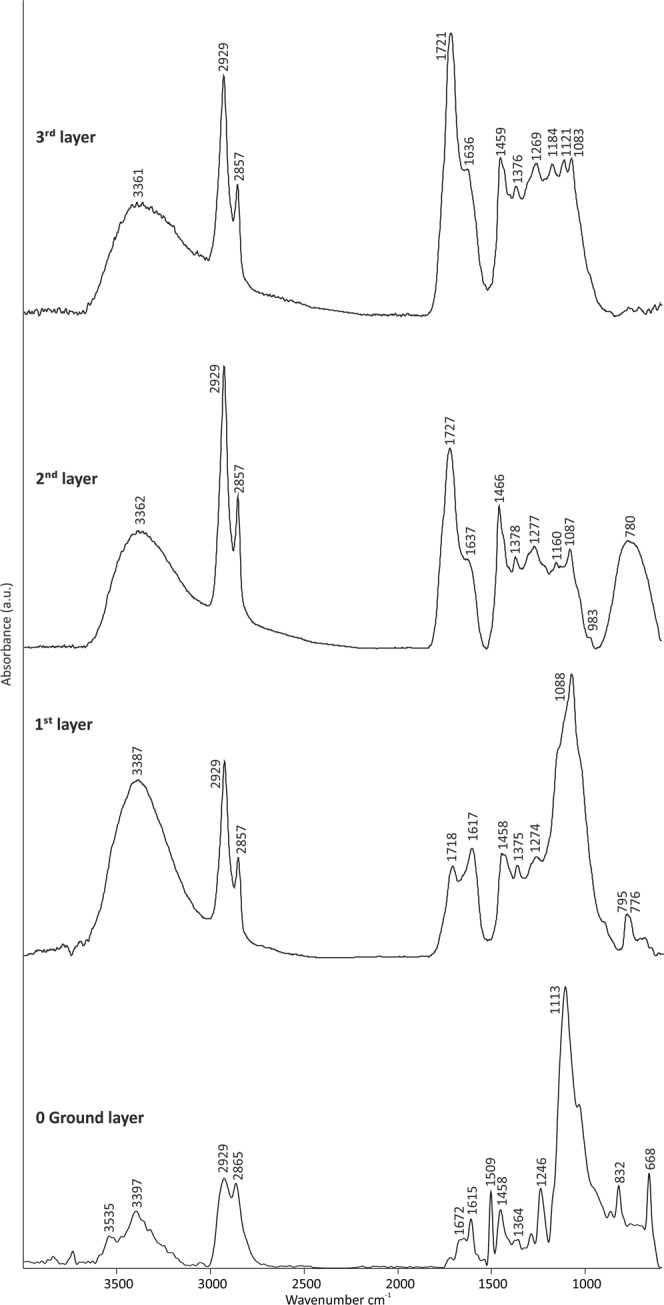
Averaged µATR-FTIR spectral profile extracted from the ground layer (0) as well as from the three painted layers of the cross-sectioned sample.

By first evaluating the averaged micro IR spectral profile extracted from each layer of the cross-sectioned sample, it was immediately possible to state that the main IR bands of the spectra collected from the upper three layers shown in Fig. 5 were related to the Vietnamese lacquer. The bands supporting this statement are the O-H stretching at 3361 cm^−1^, asymmetric and symmetric C-H stretching of CH_2_ and CH_3_ at 2929 and 2857 cm^−1^, respectively, C=O stretching at 1718 cm^−1^, C=C stretching of the polymerized catechol derivatives at approximately 1636 cm^−1^^10^, CH_2_ bending in the side chain^10,11^ at 1459 cm^−1^, CH_3_ bending at 1376 cm^−1^, C-O stretching in phenol groups and lacquer polysaccharides at 1269 and 1083 cm^−1^, respectively^10,12,13^.

The ground layer “0” was characterized by the IR bands of gypsum (CaSO_4_ ⋅ 2H_2_O) (Fig. 5) with bands at 3535 and 3397 cm^−1^ (OH stretching), 1672 and 1615 cm^−1^ (OH bending), 1113 cm^−1^ (SO_4_ stretching), and 668 cm^−1^ (SO_4_ bending). This area also exhibited a weak contribution from the epoxy resin used for embedding the sample (bands at 2929, 2865, 1509, 1458, 1364, and at 832 cm^−1^). Additionally, quartz sand (Si-O and Si-O-Si stretching at 1088 cm^−1^, Si-O stretching at 795 and 776 cm^−1^) was detected in the black Layer 1 above the ground layer, while titanium white (TiO_2_) (TiO stretching as broad band with a maximum at 780 cm^−1^) was found in the yellowish Layer 2 (Fig. 5); both of these results are consistent with the SEM-EDX results.

Unfortunately, besides the Vietnamese lacquer, these IR spectra could neither confirm nor refute the presence of the other organic compounds like linseed oil, pine resins and shellac detected by THM-GC/MS. The only indication of the presence of these other organic materials (known from the THM-CS/MS results) by µATR-FTIR are the increase in intensity and shift towards higher wavenumbers of the carbonyl band (from 1718 cm^−1^ in the first layer to 1727 cm^−1^ in Layer 2) and the more intense and sharper peaks of the CH stretching vibration at 2929 and 2857 cm^−1^ in the two upper layers. Additionally, the very weak bands at 1160 (C-O-C stretching) and 983 cm^−1^ (trans C=C-H) detected in Layer 2 (Fig. 5) could be attributed to the drying oil while a low intensity band at 1184 cm^−1^ (C-O-C stretching) in Layer 3 could be a sign of the pine resin. The main IR bands of the lacquer clearly overlap the most significant IR peaks of the other organic compounds in the same frequency regions, thus representing a challenge in distinguishing each of them in the film thickness.

Based on these observations, the application of univariate followed by multivariate analysis of the IR chemical mapping was performed for a more detailed investigation. With regards to the univariate IR chemical maps, the commonly employed integration bands function^21–26^ could not be used because of the difficulties in selecting specific marker bands of the organic compounds that were not overlapping each other, and also due to the weakness of their signal. The component regression function of the OPUS software (Bruker GmbH, Ettlingen, version 8.0) was selected as the most appropriate tool to generate IR chemical maps of the investigated sample area. This function determines the proportion of a pure component spectrum in each pixel typically containing a multicomponent spectrum. Hence, the local distribution of the pure component sample in the IR map is achieved with regards to its relative concentration. By providing a reference spectrum of the components to be analyzed, the component regression function is particularly suitable for samples where the composition is known but the distribution is unknown, as is the case in this study since the THM-GC/MS investigations already provided precise information about the organic compounds included in the sample.

**Figure 6:**
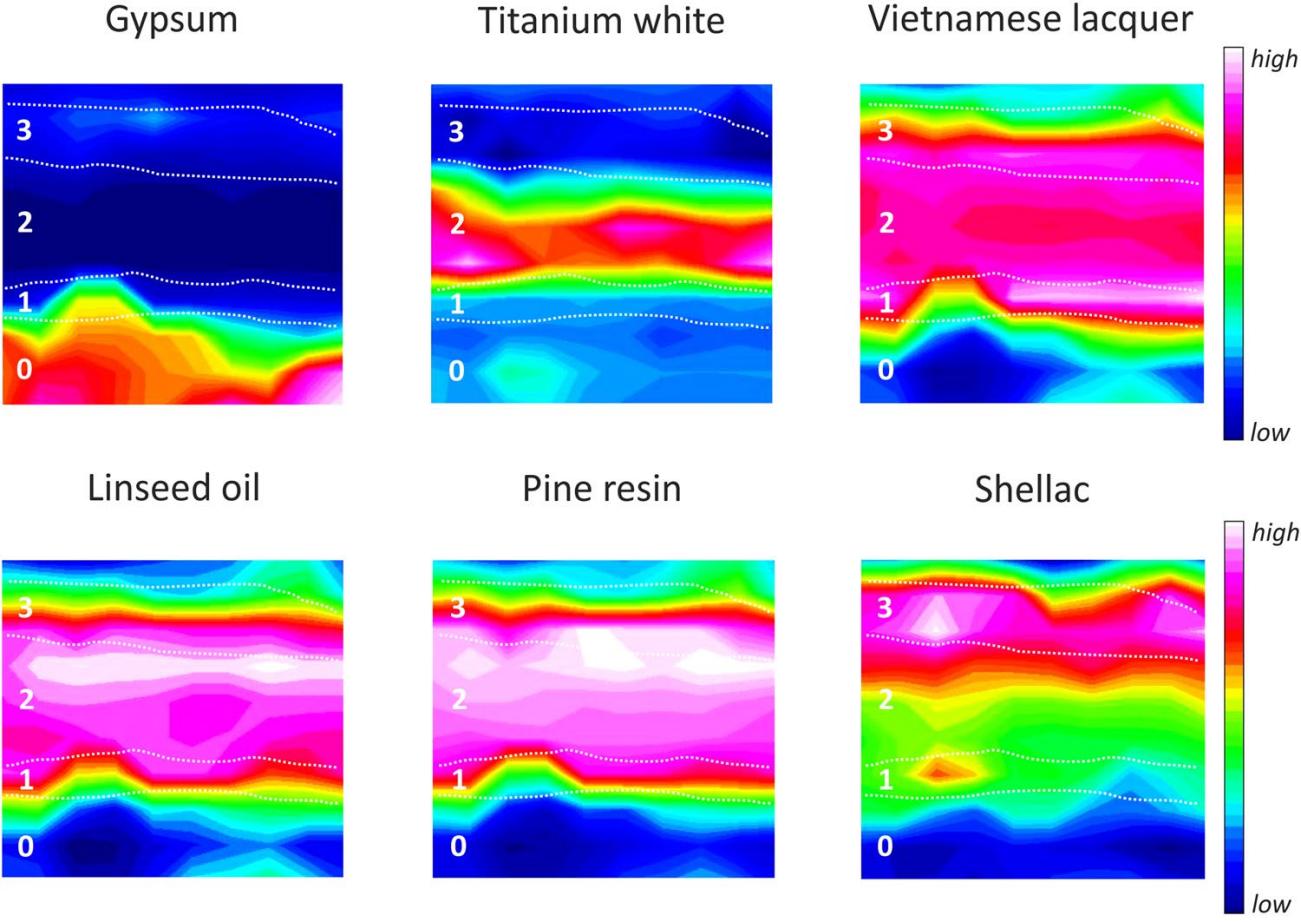
Univariate µATR-FTIR chemical maps of the main detected compounds in the cross-sectioned sample by using the component regression function.

In this way it was possible to obtain the univariate IR chemical maps depicted in Fig. 6 (color scale from white – high concentration, to blue – low concentration), which clearly show the distribution of gypsum in the ground layer (0), titanium white as part of Layer 2, and the mixture of Vietnamese lacquer with linseed oil and pine resin as additives in the upper three layers. Although Dunand developed a gypsum-based material for the ground layer, which contained lacquer^2^, the results obtained here show an absence of lacquer in the ground layer of this particular work. The IR chemical map generated for shellac, distributed most strongly at the top of the cross-sectioned sample, is particularly interesting. This latter result - especially in combination with the THM-GC/MS measurements on the superficial part of the sample (see Section 2.2) – suggests that shellac was present in Layer 3, mixed together with Vietnamese lacquer, linseed oil, pine resin and orpiment. It is existent in a high density towards the top of this layer, and continues in a slightly lower density above this layer since it was likely used as protective coating over the gilded surface or as a sealant for the gilding.

**Figure 7:**
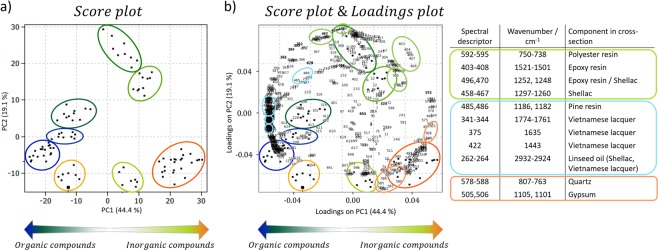
(**a**) PC1-PC2 Score plot depicting eight different groups of clusters: pale green and red at the right bottom as inorganic components, orange, light and dark blue, and the darkest green at the left side as organic components, while light and medium-dark green at the top mainly as embedding media. The localization of each cluster along the cross-section is shown in Fig. 8. (**b**) PC1-PC2 score plot superimposed with loadings plot. Loadings are marked in light green, light blue and light orange. The wavenumbers corresponding to the marked spectral descriptors in the loadings plot are listed up with the according assignment to a component in the cross-section.

Principal Component Analysis (PCA) as unsupervised, multivariate analysis of the IR spectral dataset was used to confirm the spatial distribution of the main organic elements in the lacquer cross-section already detected by the univariate chemical imaging. PCA is a linear transformation of the coordinate system of an n-dimensional dataset in which variance is exhibited by data points. Hence, it allows the reduction of the complexity of a dataset by reducing its dimensionality. As the PC1-PC2 score plot in Fig. 7a illustrates, eight different clusters can be distinguished. PC1 differentiates between the two inorganic compounds found in the lower part of the paint cross-section (lower right side of the PC1-PC2 score plot) and the four organic components attributed to the upper layers (left side of the PC1-PC2 score plot). Additionally, PC2 allows the differentiation of layers containing the embedding paint media from the organic and inorganic components in the cross-section of the painting.

**Figure 8:**
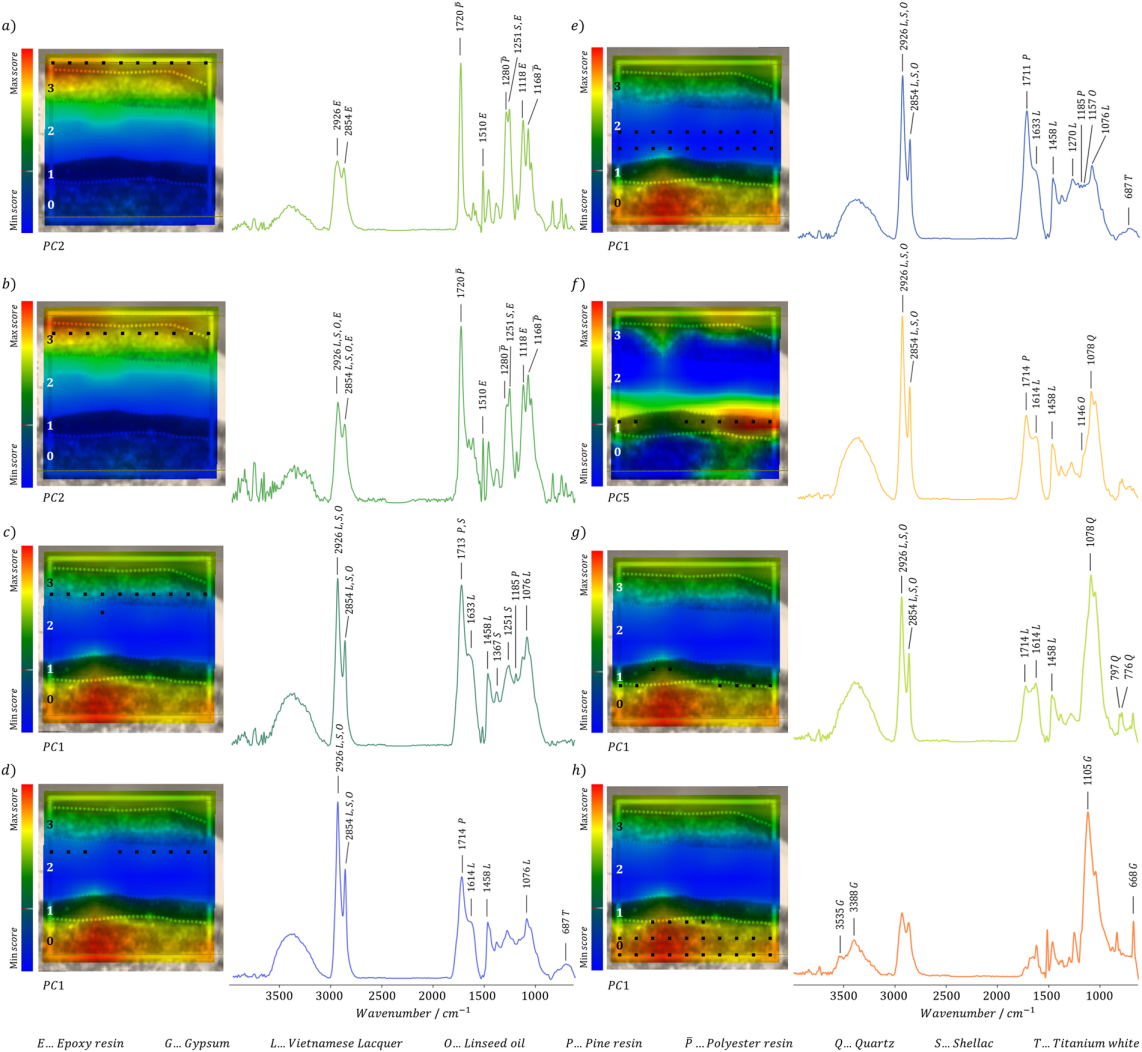
PC score maps with the respective average spectral profiles (positions marked with black squares) extracted from each paint layer with the bands of the main components marked in the spectra.

These observations are sustained by the evaluation of their correspondence with the loadings located in the matching directions (Fig. 7b) as well as by the extracted average profile of the acquired spectra. Furthermore, the visualization of the clusters in the score maps of PC1, PC2, and PC5, respectively, shown in Fig. 8, allowed the determination of their position along the paint layers. Based on this assignment, it is possible to confer the red and light green clusters to gypsum and quartz, distributed in the ground Layer 0 and Layer 1 respectively, according to their average spectral profile (Fig. 8g,h).

On the other hand, the four clusters in the lower left part of the PC1-PC2 score plot can be assigned to four different areas amongst the paint Layers 1, 2, and 3 (Fig. 8c–f): the darkest green colored cluster represents the Layer 3 (Fig. 8c), dark and light blue the upper and lower part of the Layer 2 (Fig. 8d,e), and light orange cluster represents the paint Layer 1 (Fig. 8f). According to the evaluation of the loadings and extracted average spectral profiles, their differentiation is mostly attributable to the C-O-C fingerprint region (1260–1100 cm^−1^) with a dominating influence of the Vietnamese lacquer (2926, 2854,1633–1614, 1458, 1270, and 1076 cm^−1^), which is best visible in the light and dark blue clusters located at the upper and lower part of Layer 2 (Fig. 8e,f). The IR band at 1185 cm^−1^ suggests the presence of pine resin (Fig. 8d) in the blue-labeled cluster in the upper part of Layer 2. This band is also visible in the mean spectrum of the lower part of the Layer 2 and might still be present in traces in Layer 1. Bands at 1157–1146 cm^−1^ indicate the presence of linseed oil in the lower part of the Layer 2 (Fig. 8e) and Layer 1 (Fig. 8f). Additionally, Layer 1 also contains a prominent spectral feature attributed to quartz (1178 cm^−1^, Fig. 8f), which is even more pronounced in the spectrum describing the lower part of this layer (Fig. 8g). Finally, the band at 1251 cm^−1^ proposes the presence of shellac in the darkest green labeled cluster of the lower part of the paint Layer 3 (Fig. 8c). Hence, these data indicate the existence of Vietnamese lacquer mixed with linseed oil and pine resin in the upper three layers and also the addition of shellac in Layer 3, thus confirming the information previously extracted by the univariate analysis of the µATR-FTIR chemical map. The presence of gypsum in Layer 0 is confirmed by the cluster colored in red depicting typical IR bands attributed to gypsum, such as the OH stretching vibrations at 3535 cm^−1^ and 3388 cm^−1^, the SO_4_ stretching vibration at 1105 cm^−1^ and the SO_4_ bending vibration at 668 cm^−1^. Furthermore, the two clusters localized in the upper half of the PC1-PC2 score plot (Fig. 7a) mainly contain spectral features attributed to the embedding media (polyester and epoxy resin), which primarily affects the results from the upper part of the cross-section (Fig. 8a,b).

Concerning the coating/protective fifth layer with a strong bluish fluorescence (Fig. 2b) individuated by OM in UV light (Section 2.1), neither the univariate nor the multivariate chemical imaging allowed the detection or identification of this layer. Nevertheless, point-IR analysis performed on an additional sample of this transparent top layer, with remnants of the attached lower layer, allowed the characterization of this coating material. The obtained IR spectrum showed the overlapping three main bands of nitrocellulose at 1646 (NO_2_ asymmetric stretching), 1279 (NO_2_ symmetric stretching), and 850 cm^−1^ (CH_2_ rocking vibration) (see Supplementary Information, Figure S-1) in the paint spectrum, as well as the main bands of shellac, thus confirming their presence in the upper part of the lacquered panel painting. Nitrocellulose-based varnish appears to have been used on “The Return of the Hunters” to seal the gilding during later reworking of the panels between 1962 and 1992^3^.The existence of nitrocellulose was also confirmed for this study by the use of a diphenylamine test, the method of which is published elsewhere^27^.

## Conclusions

The scientific investigations on “The Return of the Hunters” by Jean Dunand presented here revealed a great deal of information about the materials and artistic techniques used by the artist. In particular, THM-GC/MS identified the type of binder used by Dunand - not just Vietnamese lacquer alone - but lacquer in combination with organic additives like linseed oil, pine resin, and shellac. The OM in Vis and UV light showed a total of five paint layers characterized by different types of inorganic pigments including titanium white, bone white, orpiment, vermillion, and a final application of gold and copper particles, all confirmed by SEM-EDX. Furthermore, this work showed how the results obtained by THM-GC/MS permitted the assignment of different organic compounds to specific layers in a highly complex lacquer paint stratigraphy as determined by univariate and then multivariate analysis of chemical mapping µATR-FTIR. This was employed as a novel approach for the investigation of lacquered paint samples allowing getting a more detailed picture on the spatial distribution of the several and different components along the cross-section of the lacquered wall-panel. It was possible to visualize the application of Dunand s favorite “*Laque arrachee*” technique which used Vietnamese lacquer mixed with linseed oil, pine resin, and quartz to form a black, compact and homogenous layer with a wavy surface applied on a plaster ground layer made of gypsum with red iron particles as coloring material. After the black lacquer layer, a pale yellow layer made of Vietnamese lacquer mixed with linseed oil and pine resin as well as titanium white and bone white pigments was applied. The lacquer layer was composed of Vietnamese lacquer mixed with the organic compounds linseed oil, pine resin and shellac. This layer included the pigments orpiment and vermillion, which imparted a dark orange color to the lacquer. Additionally, the obtained results showed that Dunand used both of gold and copper metals for assigning different finishing color effects to the paint surface, and that nitrocellulose was later applied as coating/varnish.

The knowledge gained about the materials and techniques used by Dunand by means of this comprehensive multi-analytical approach were then employed to form an appropriate conservation-restoration strategy. After knowing the main chemical composition of the original layers, solubility tests applied on larger areas of the painting including the other panels helped to distinguish with certainty the revised zones from the originals. Moreover, this information also documents one of the first examples of the use of Vietnamese lacquer as a painting medium in Europe, a significant moment in the course of art history.

## Methods

Four samples taken from lacquer panel number three (100 cm × 124.5 cm) of “The Return of the Hunters”, were the subject of analysis. The first sample, named P3_UL, corresponded to the ground layer gypsum together with some paint layers and was investigated by THM-GC/MS for determining the type of lacquer and other organic compounds. The second sample, labeled P3_OL, was taken from the upper layers of the object, and was analyzed to determine the possible presence of shellac. The third specimen (P3_L) corresponding to the whole paint stratigraphy was used for making a cross-section, and was studied with OM in Vis and UV light, SEM-EDX, as well as with µATR-FTIR mapping. Finally, the fourth sample P3_U was the coating/protective layer, which was investigated by µATR-FTIR. The scientific investigations used for this research study are described in detail in the Supplementary Information section.

## Supplementary information

Supplementary Information

## Data Availability

The authors of the article entitled “Comprehensive Multi-Analytical Investigations on the Vietnamese lacquered Wall-Panel “The Return of the Hunters” by Jean Dunand” Valentina Pintus, Anthony J. Baragona, Karin Wieland, Michael Schilling, Silvia Miklin-Kniefacz, Christoph Haisch, and Manfred Schreiner – declare hereby data availability.
