# Supplementary material for: Comprehensive Multi-Analytical Investigations on the Vietnamese lacquered Wall-Panel “The Return of the Hunters” by Jean Dunand
Source: Sci Rep. 2019 Dec 11;9:18837. doi: 10.1038/s41598-019-55471-6 (PMC6906402; doi:10.1038/s41598-019-55471-6)
Supplement: Supplementary file 1 — Supplementary Information [file 41598_2019_55471_MOESM1_ESM.pdf]

Comprehensive Multi-Analytical Investigations on the Vietnamese lacquered Wall-Panel "The Return of the Hunters" of Jean Dunand

Valentina Pintus[1], Anthony J. Baragona[2], Karin Wieland[3], Michael Schilling[4], Silvia Miklin-Kniefacz[5], Christoph Haisch[3], Manfred Schreiner[1]

1. Institute of Science and Technology in Art, Academy of Fine Arts Vienna, Schillerplatz 3, Vienna, A-1010, Austria
2. Institute of Art and Technology, Department of Conservation Science, University of Applied Arts, Salzgries 14, A-1010 Vienna, Austria
3. Chair of Analytical Chemistry, Institute of Hydrochemistry, Technical University of Munich (TUM), Marchioninistraße 17 D-81377 Munich, Germany
4. Getty Conservation Institute (GCI), Los Angeles, CA, U.S.A.
5. Bernardgasse 4/1, Vienna, A-1070, Austria

Corresponding author´s email: v.pintus@akbild.ac.at

Optical microscopy (OM) and scanning electron microscopy with energy dispersive X-ray spectroscopy (SEM-EDX)

The stratigraphy of a cross-sectioned sample named P3_L was studied with both, OM and SEM-EDX. To prepare the cross-section, a tiny and representative fragment was taken from the selected sample and placed on a dry layer of polyester resin mixed with 1 % hardener (type GTS, VOSSCHEMIE, Germany) and finally embedded into epoxy resin (type Araldite 2020/A) with 3 % hardener (type 2020/B, Huntsman, U.S.A.) resin. In order to observe the stratigraphy under the OM and SEM without any interference of a rough surface, the cross-section was polished by using abrasive grinding paper based on silicon carbide (SiC) with different grit sizes (from 600 to 12000 grit) after drying/hardening for 5 days at room temperature.

For the optical microscopy a ZEISS Axioplan 2 Imaging microscope (10x, 20x, 50x objectives and 10x oculars) was employed by using visible (Vis) as well as UV-radiation (UV lamp HBO 100). The images of the sample were acquired by a Nikon D700 Camera and evaluated with the Camera Control Pro2 Software. SEM-EDX analysis was performed using a Quanta FEG 250 (FEI, U.S.A.) scanning electron microscope coupled to the Octane Elect Plus EDX detector (Ametek/EDAX, U.S.A.) equipped with the Genesis EDX Quant software. The investigations were performed under low vacuum at an accelerating voltage of 20 kV in back scattered electron (BSE) detection mode.

Thermally assisted hydrolysis and methylation – gas chromatography / mass spectrometry (THM-GC/MS)

For the THM-GC/MS analysis specimens of approximately 0.22 mg were taken from the P3_UL and P3_OL samples and put in a sample cup (ECO-CUP Frontier Lab, Japan). Each

of them was subsequently treated with 2 µL tetramethylammonium hydroxide (TMAH) reagent (25 wt% aqueous solution of TMAH, Sigma-Aldrich, U.S.A.). For the analysis of the samples the pyrolyzer PY-2020iD (Frontier Lab, Japan) combined with a GCMS-QP2010 Plus (Shimadzu, Japan) was employed. The GC/MS unit was equipped with a capillary column SLB-5ms Supelco, U.S.A. (30 m length x 0.25 mm internal diameter x 0.25 µm film thickness) with bonded and highly cross-linked 5% diphenyl / 95% dimethyl siloxane as stationary phase. The capillary column was connected with a deactivated silica pre-column Rxi Guard Column Restek, U.S.A. (5 m length x 0.32 mm internal diameter).

For both, Py-GC/MS and THM/GC-MS analysis, the pyrolysis temperature was set to 500 °C, while the pyrolysis interface and the injector temperature were set to 280 °C and 250 °C, respectively. THM-GC/MS analysis was performed using 2 µl of TMAH methylating reagent (25 wt% aqueous solution of TMAH, Sigma-Aldrich, USA), which was added in a sample cup (ECO-CUP Frontier Lab, Japan) containing a fraction of the P3 sample (around 0.22 mg). The GC column temperature conditions were set as follows: Initial temperature 40 °C, held for 2 minutes and followed by a temperature increase of 6 °C/min to 300 °C and held for 20 minutes. The helium gas flow was set to 1 mL/min and the electronic pressure control was set to a constant flow of 31.7 ml/min, in split mode at a 1:50 ratio. The mass spectra were recorded under electron impact (EI) ionization in positive mode at 70 eV and the temperature of the MS interface and the ion source were 280 °C and 200 °C, respectively. The mass spectrometer was scanned from m/z 50 to m/z 750. For the THM-GC/MS a solvent cut time of 5 min by turning off the filament in the ion source was used. This mode prevents the sharp decrease of the vacuum inside the ion source due to the entrance of the TMAH reagent, which has a detrimental effect on the filament and other components.

The processing and evaluation of the obtained results was performed by using an expert system based on the combination of ADMIS (Automated Mass Spectral Deconvolution & Identification System) and a specialized Excel workbook, respectively. This expert system has been developed by the Getty Conservation Institute (GCI, Los Angeles, USA) and the J. Paul Getty Museum (JPGM) and is described in detail elsewhere [3].

Micro attenuated total reflection of Fourier transform infrared spectroscopic (µATR-FTIR) mapping

µATR-FTIR analyses were performed with a LUMOS Standalone FTIR microscope (Bruker Optics GmbH) equipped with a Globar thermal light source, a RockSolid^{Tm} interferometer, and a liquid nitrogen cooled mid-band 100 x 100 µm^2 Photoconductive Mercury Cadmium Telluride (PC-MCT) detector. The ATR probe was a germanium frustum cone-shaped crystal (Ge, refractive index *n*=4) with a tip diameter of 100 µm. This ATR probe is implemented into a fully motorized and automated piezo motors 8x Cassegrain objective (NA=0.6). A XYZ motorized

sample stage allows selecting a priori the applied pressure of the ATR probe during the measurements in three different modes such as low, medium, and high. Due to the sensitivity of the cross-section material a low pressure was selected, which did not leave any microscopically recognizable print on the surface of the sample. All the optics and beamsplitter were made of zinc selenide (ZnSe). Spectra were acquired in the spectral range between 4000 and 600 cm^{-1}, performing 128 scans at 4 cm^{-1} resolution. The resulting spectra were collected and evaluated with the spectrum software OPUS-IR™ (Bruker Optics GmbH, Version 8.0).

Details about the IR mapping performed on the cross-sectioned sample are reported in the Results and Discussion section. Univariate analysis of the IR map was performed employing the component regression function implemented in the OPUS software.

Principal component analysis (PCA) for unsupervised multivariate analysis of chemical maps

Principal Component Analysis (PCA) of the µATR-FTIR spectral dataset was performed on the standardized data (mean = 0, standard deviation = 1) using ImageLab software (Epina GmbH, Retz, Austria; Version 2.93). Analysis was based upon 629 spectral descriptors (intensity value at wavenumbers spaced apart 4 cm^{-1} corresponding to the spectral resolution of the IR map) including the spectral region from 4000-2736 cm^{-1} and 1900-600 cm^{-1}. Prior to PCA, the spectra were processed in OPUS software according to the following steps: The asymmetric CO_2 stretching mode was eliminated by straight line generation between 2450 and 2200 cm^{-1} to facilitate data processing. Concave rubberband correction (6 iterations, 30 baseline points) was employed for baseline correction and vector normalization was applied for better comparison between single spectra.

µATR-FTIR spectrum of coating/protective layer

The µATR-FTIR spectrum of the coating/protective layer was collected by single measurement spots along the isolated layer from the sample named P3_U. Figure S-1 shows the spectral overlap of three main bands of nitrocellulose (1646, 1279, and 840 cm^{-1}) with the measured sample spectrum, which otherwise exhibits strong spectral similarity to the shellac reference spectrum. Hence, the presence of nitrocellulose as coating/protective layer and also of shellac, both as main components of the upper part of the lacquered panel painting, can be confirmed based on IR spectroscopic data.

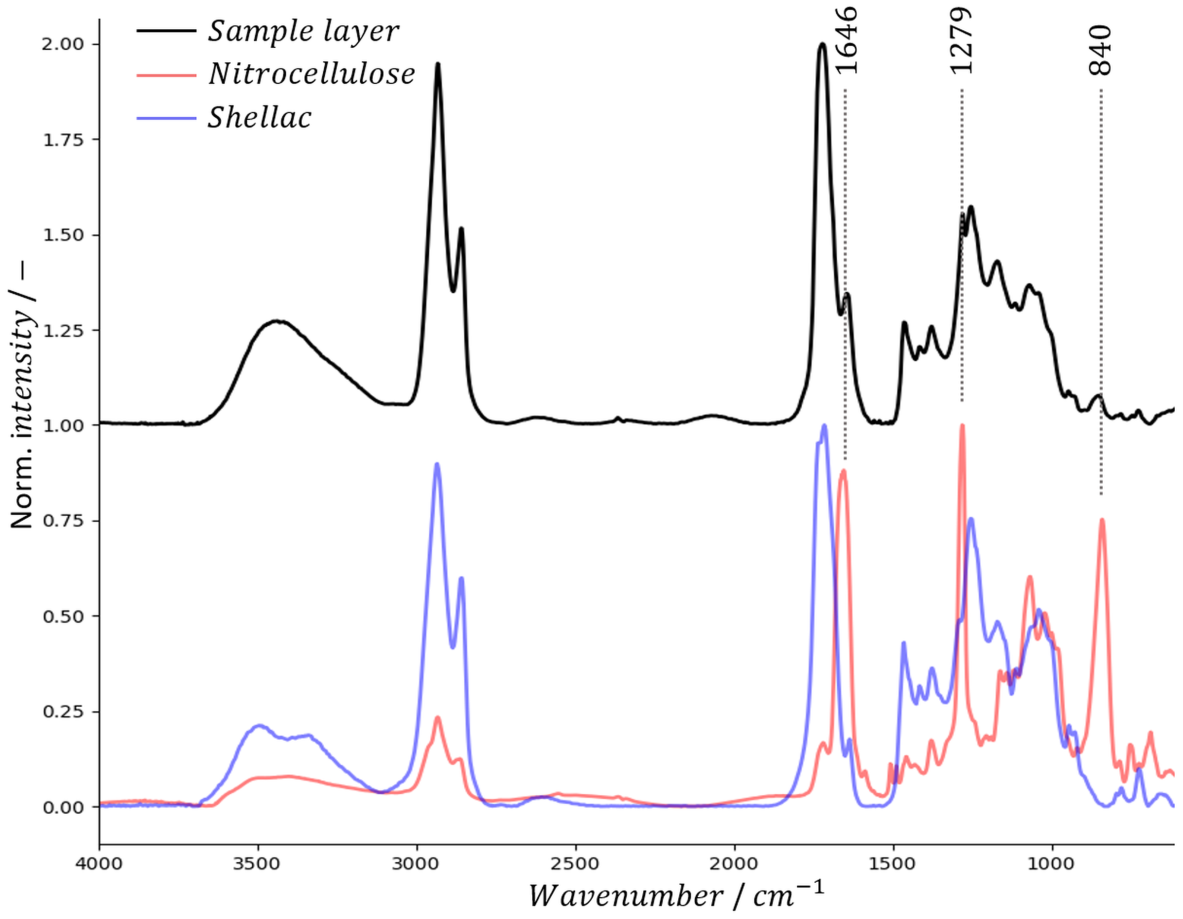

Figure S-1: µATR-FTIR spectra of the coating/protective layer (black line) compared to the reference spectra of nitrocellulose (red line) and shellac (blue line) taken from the IRUG library. The three most intense nitrocellulose bands are marked by grey dotted lines.
